# In Situ Measurements of Domestic Water Quality and Health Risks by Elevated Concentration of Heavy Metals and Metalloids Using Monte Carlo and MLGI Methods

**DOI:** 10.3390/toxics10070342

**Published:** 2022-06-21

**Authors:** Delia B. Senoro, Kevin Lawrence M. de Jesus, Ronnel C. Nolos, Ma. Rowela L. Lamac, Khainah M. Deseo, Carlito B. Tabelin

**Affiliations:** 1 Resiliency and Sustainable Development Laboratory, Yuchengco Innovation Center, Mapua University, Intramuros, Manila 1002, Philippines; klmdejesus@mymail.mapua.edu.ph (K.L.M.d.J.); rcnolos@mapua.edu.ph (R.C.N.); kmdeseo@mapua.edu.ph (K.M.D.); 2 School of Graduate Studies, Mapua University, Intramuros, Manila 1002, Philippines; 3 School of Chemical, Biological, Materials Engineering and Sciences, Mapua University, Intramuros, Manila 1002, Philippines; 4 School of Civil, Environmental, and Geological Engineering, Mapua University, Intramuros, Manila 1002, Philippines; 5 Mapua-MSC Joint Research Laboratory, Marinduque State College, Boac 4900, Philippines; wellallamac@gmail.com; 6 Department of Environmental Science, College of Natural and Allied Health Sciences, Marinduque State College, Boac 4900, Philippines; 7 School of Minerals and Energy Resources Engineering, University of New South Wales, Sydney 2052, Australia; c.tabelin@unsw.edu.au

**Keywords:** carcinogenic risk, domestic water, machine learning, metal pollution, spatial distribution maps

## Abstract

The domestic water (DW) quality of an island province in the Philippines that experienced two major mining disasters in the 1990s was assessed and evaluated in 2021 utilizing the heavy metals pollution index (MPI), Nemerow’s pollution index (NPI), and the total carcinogenic risk (TCR) index. The island province sources its DW supply from groundwater (GW), surface water (SW), tap water (TP), and water refilling stations (WRS). This DW supply is used for drinking and cooking by the population. In situ analyses were carried out using an Olympus Vanta X-ray fluorescence spectrometer (XRF) and Accusensing Metals Analysis System (MAS) G1 and the target heavy metals and metalloids (HMM) were arsenic (As), barium (Ba), copper (Cu), iron (Fe), lead (Pb), manganese (Mn), nickel (Ni), and zinc (Zn). The carcinogenic risk was evaluated using the Monte Carlo (MC) method while a machine learning geostatistical interpolation (MLGI) technique was employed to create spatial maps of the metal concentrations and health risk indices. The MPI values calculated at all sampling locations for all water samples indicated a high pollution. Additionally, the NPI values computed at all sampling locations for all DW samples were categorized as “highly polluted”. The results showed that the health quotient indices (HQI) for As and Pb were significantly greater than 1 in all water sources, indicating a probable significant health risk (HR) to the population of the island province. Additionally, As exhibited the highest carcinogenic risk (CR), which was observed in TW samples. This accounted for 89.7% of the total CR observed in TW. Furthermore, all sampling locations exceeded the recommended maximum threshold level of 1.0 × 10^−4^ by the USEPA. Spatial distribution maps of the contaminant concentrations and health risks provide valuable information to households and guide local government units as well as regional and national agencies in developing strategic interventions to improve DW quality in the island province.

## 1. Introduction

The fundamental requirement for human growth and development is water. Around 71% of the world’s population relies on a clean drinking water supply that is readily available and free of contaminants, but 29% of the population still does not have safe and uncontaminated water access. Over 785 million people, including 144 million who depend on surface water, do not have access to a primary source of drinking water [1]. Accelerated population growth, social and urban development, and industrialization all place enormous demands on water resources, leading to an increased risk of contamination and depletion in the future [2]. Natural geological factors and human-induced activities often affect the quality of a region’s water resources. The presence of heavy metals and metalloids (HMM) in water resources, for example, could be caused by natural phenomena such as weathering, oxidation, water flow direction, topographical characteristics, hydrological processes, and diverse rock types. Similarly, human activities impact water resources through rapid growth in population, economic development, and improper management of mining operations [3].

The HMM contamination of water resources is linked to increased toxicity, persistence, and resistance to degradation, posing a threat to humans and causing substantial health concerns [4]. In the Philippines, domestic water (DW) is commonly sourced from surface water (SW) and groundwater (GW). Water distribution systems are standard where water from a centralized treatment plant, tanks, or wells is brought to consumers free or with a charge. In addition, hand pump wells, often utilized in rural regions for domestic purposes, are used to extract GW [5]. A fraction of the population has also inclined to substitute drinking water resources from refilling stations [6]. However, anthropogenic activities such as mining, which are not well regulated and managed, have increased the possibility of irreversible contamination and deterioration of SW and GW resources [7]. Activities associated with mining operations such as mineral extraction, smelting and refining, and tailings disposal could be a potential source of HMM pollution in the environment [8].

The Marinduque province in the Philippines is known for its abundant metallic and nonmetallic deposits and is notable for having significant porphyry copper resources [9]. Between 1969 and 1996, the island was a site for mining operations [10]. The island province was devastated by two mining catastrophes deemed two of the world’s most catastrophic mining disasters. Since then, two abandoned open mine pits on the island, the Tapian and the San Antonio mine pits, remained unrehabilitated and continue to adversely affect the island’s environmental quality and water resources [11]. High concentrations of HMMs were observed across the island in various media such as water and soils [12], SW and sediments [10,13], agricultural food crops [11], and freshwater crustaceans and tilapia [14].

Although previous studies examined specific HMM and locations within the island province, no comprehensive evaluation of HMM pollution and health risks (HR) linked to GW resources has been conducted. Numerous rating systems were developed to assess the water quality (WQ) and human HR instigated by HMM through exposure to elevated HMM concentration [15]. The degree and impact of HMMs as they enter the human body are quantified using health risk (HR) indicators. These indices include the hazard index, which determines the overall risk of noncarcinogenic consequences caused by several chemicals. It is determined using the chronic daily intake (*CDI*) and the reference dose (*RfD*). It is noteworthy that many metals and metalloids are necessary for human health; however, some of them are toxic even in trace concentration [16]. Toxic HMM deposits in the environment can be dangerous to the population if not adequately controlled and managed [14]. Humans are in greater danger from HMM since they may enter the body through the food chain and various pathways with possible carcinogenic effects [17]. Hence, special attention should be focused on the health concerns posed by HMM.

Risk assessment (RA) is a technique to determine the probable effects of pollutants on target receptors such as humans. It is divided into two main categories: (i) RA for human health, and (ii) RA for the environment [18]. Every RA of water quality (WQ) will always have some uncertainty; thus, doing an uncertainty analysis as part of the RA process would assist in more accurately identifying and analyzing target areas and environmental compartments for better intervention and control [17].

The Monte Carlo simulation (MCS) has been recognized by the United States Environmental Protection Agency (USEPA) as a practical approach for uncertainty analysis of an RA, and it is frequently used for HMM RAs. RAs for such environmental compartments have become more significant as the levels of HMM in water supplies have increased [19,20]. Marinduque, which hosted catastrophic mining disaster events, has a limited study on human HR analysis to exposure to HMM in the DW. Most of the population relies on SW and GW for DW supply. Moreover, some water refilling stations (WRS) used SW and GW as the source of water for their bottled and refilling business. Hence, an RA considering uncertainty analysis and probabilistic HR evaluation methodologies such as MCS is essential.

Regarding the stated difficulties of HMM contamination of water resources, in situ, precise, and real-time detection of HMMs in water are needed. Laboratory-based methods such as inductively coupled plasma optical emission spectroscopy (ICP-OES) and atomic absorption spectroscopy (AAS) have drawbacks, as these methods need several days to complete the analyses. These laboratory analytical techniques are also costly, involve complicated sample preparation, and are inapplicable in field environments [21]. The utilization of portable X-ray fluorescence (pXRF) and metals analysis systems (MASs) for in situ metals detection addresses these limitations by providing a speedy and reliable analysis. This in situ HMM detection and analysis, coupled with the MCS and mapping, produces a rapid and reliable RA. Hence, the primary purpose of this research was to provide new knowledge to the population of the quality of their DW supply, the available onsite detection, and analysis technology. The specific objectives of this study were to: (1) assess HMM pollution at specific sites using various indices such as *MPI* and *NPI*; (2) evaluate the carcinogenic and noncarcinogenic HRs with the application of MCS to analyze the uncertainty in the results; and (3) create a machine learning—geostatistical interpolation (MLGI)—based spatial interpolation maps of pollution and health risk index of DW in Marinduque. The significance of this study is the provision of benchmark data for local DW quality monitoring specifically the HMM concentrations and the health risks to go along with the DW supply quality. In addition, this information provides the groundwork for the development, utilization, and protection of water resources.

## 2. Materials and Methods

### 2.1. Study Area

**Figure 1:**
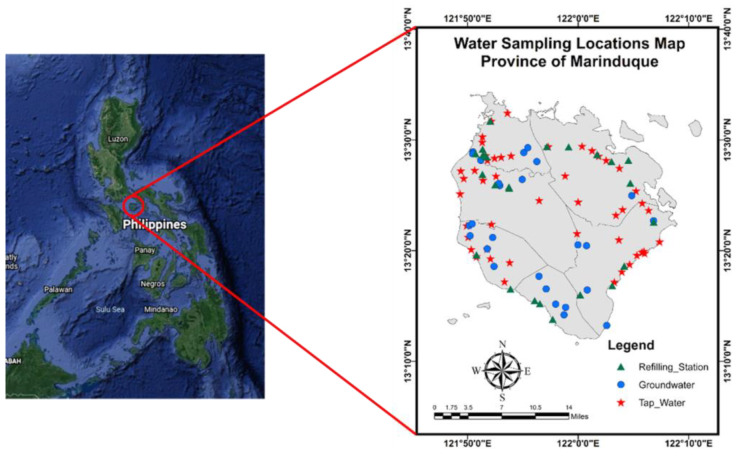
A map of the Philippines showing Marinduque Island as a zoomed-in inset, including domestic water sampling locations for this study.

The DW samples were gathered across the island province of Marinduque, Philippines, as shown in Figure 1. Marinduque is located about 200 km south of Manila, the capital city of the Philippines, covering about 96,000 hectares of land [22]. It comprises six municipalities: Boac, Buenavista, Gasan, Mogpog, Sta. Cruz, and Torrijos. It is a tropical and warm island with an annual mean temperature of 23 to 28 °C [23]. The geology of the island is characterized by permeable volcanic and sedimentary rocks, which allow groundwater to flow through across the island [14,24].

### 2.2. Water Samples Collection, Preparation, and Analysis

**Figure 2:**
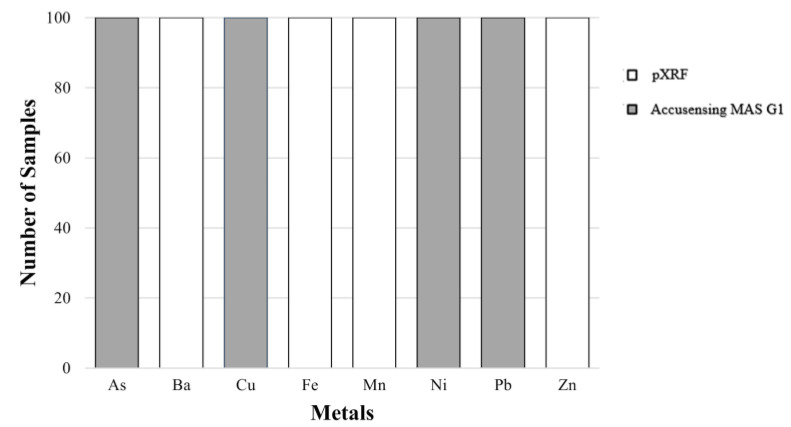
Analysis of HMMs using Accusensing MAS G1 and pXRF.

One hundred grab samples in a 500 mL volume were collected using the suggested standard procedure [25]. Twenty-five water samples from WRS, twenty-six groundwater (GW) samples, and twenty-one tap water (TW) samples were collected and placed in polyethylene (PE) bottles. All water samples were either used for drinking and/or used for cooking by households. The PE bottles were washed with distilled water or Type 1 water [26] to remove contaminants. Physicochemical parameters such as temperature, pH, total dissolved solids (TDS), and electrical conductivity (EC) were evaluated and recorded in situ using a Hannah Multiparameter HI 9811-5 portable meter [27]. The portable handheld Olympus Vanta X-ray fluorescence spectrometer and Accusensing Metals Analysis System (MAS) G1 were utilized for the HMM concentration analysis. Both analyzers are high-performance and on-site elemental analyzers that may be used with various environmental media, including water. The pXRF spectrometer was calibrated using the Olympus Vanta blank and set to Geochem mode before analysis [21]. The Accusensing MAS G1 was used for analyzing HMMs which registered “limits of detection” (LOD) in the pXRF such as As, Cu, Ni, and Pb. Figure 2 shows the metals analyzed using the two analyzers.

### 2.3. HM Pollution and HR Assessment

The detected elements in the water samples were assessed and compared to the Philippine National Standards for Drinking Water (PNSDW) 2017 [28]. A HMM pollution assessment utilizing several indices such as the *MPI*, *NPI*, non-CR Index, and CR Index was implemented in this study to comprehensively assess the degree of pollution in the DW considering HMMs.

#### 2.3.1. HMM Pollution Index (*MPI*)

The *MPI* describes the WQ state and determines whether it is suitable for drinking concerning HMMs. The *MPI* is established on the weighted arithmetic mean quality procedure as presented in Equation (1).

(1) $$MPI=\frac{\sum_{i=1}^{n}Q_{i}W_{i}}{\sum_{i=1}^{n}W_{i}}$$

where *W_i_* is denoted as the weight unit, which can be computed as 1/*S_i_* where *S_i_* is the suggested level of the pertinent HMM, “*n*” is the value of the evaluated HMMs, and *Q_i_* is the distinctive quality rating of the “*i*th” metal and specified by Equation (2).

(2) $$Q_{i}=\frac{C_{i}}{S_{i}}\times 100$$

*C_i_* is the detected value of the *i*th metals in micrograms/liter. The standard allowable value (*S_i_*) for each constraint was acquired from the Philippine water quality standards [29]. The categorization of the WQ using *MPI* is demonstrated in Table 1.

#### 2.3.2. Single-Factor Pollution Index (*SFPI*)

The Single Factor Pollution Index (*SFPI*) was employed to calculate and describe the effect of an individual HMM as it contaminates the DW at a specific sampling location. The *SFPI* is computed using Equation (3) presented below:

(3) $$SFPI=\frac{C_{i}}{S_{i}}$$

where *C_i_* denotes the observed intensity of the pollutant “*i*” in the groundwater (in mg/L) and *S_i_* is the evaluation standard of the contaminant “*i*” in the surface and groundwater (in mg/L). An *SFPI* value greater than 1 signifies that the heavy-metal pollutant exceeds the standard [31].

#### 2.3.3. Nemerow’s Pollution Index (*NPI*)

The *NPI* was utilized to calculate and explain the influence of several HMMs as they pollute the water resource at a particular sampling location. The *NPI* is the most usual method and thorough pollution assessment approach that reflects the single factor index *P_i_*, highlights the impact of elevated levels of HMMs on the environmental quality, and removes the insufficiency of the mean value on assessment. The *NPI* is calculated using Equation (4), shown below:

(4) $$NPI=\sqrt{\frac{{\left(SFPI_{\max}\right)}^{2}+{\left(SFPI_{ave}\right)}^{2}}{2}}$$

where *SFPI*_max_ indicates the highest *SFPI* of a pollutant and *SFPI_ave_* denotes the mean *SFPI* of the pollutants considered [32,33]. The classification of *NPI* values is shown in Table 2.

#### 2.3.4. Non-CR and CR Index

HMMs are introduced into the body by various pathways, with ingestion—the most frequent route of water consumption—as a common pathway resulting from oral human exposure. The *CDI* quantifies the extent of pollution absorbed by the human body and specifies the pollutant dosage per kilogram of body weight per day as received by direct eating, dermal absorption, or inhalation, as suggested by the USEPA. The *CDI* of water ingestion can be calculated using Equation (5) [35]:

(5) $$CDI_{in}=\frac{c_{i}\times IR\times EF\times ED}{BW\times AT}$$

where *CDI_in_* is the exposure doses from ingestion of water in gram/kilogram-day and *C_i_* is the mean concentration of the *i*th HMM in water (micrograms/liter) [36]. Additional amounts and units of other parameters in the computation of *CDI* are presented in Table 3.

The relevant *RfD* was compared to the exposure or mean intake of hazardous elements to determine the probability on noncarcinogenic substances. The noncancer risk was quantified using the hazard quotient (*HQ*) for a single chemical or the hazard index (*HI*) for multiple substances and exposure routes. Concerns about possible noncarcinogenic consequences may arise if exposure to a chemical exceeds the corresponding *RfD*, i.e., if *HQ* exceeds 1 [43] as shown as Equation (6).

(6) $$HQ=\frac{CDI}{RfD}$$

Table 4 presents the toxicity reactions of HMMs for *RfD* values for both oral and dermal exposure route as well as the oral slope factor (*SF*).

The total potential for non-CRs influenced by calculating chemicals can be evaluated by the *HI*, which is the sum of each computed *HQ*. Equation (7) displays the formula for calculating the hazard index.

(7) HI=∑HQ

It is recommended to have even greater probabilities of harmful health effects when the *HI* is greater than 1. At the same time, no chronic risks were expected to transpire at the site when *HI* was less than 1 [48].

The USEPA defined *CRs* as the cumulative risk of a person developing cancer because of exposure to a probable cancer-causing agent. The cancer risk was calculated by Equation (8):

(8) CR=CDI×SF

The total cancer risk (*TCR*) is the sum of the cancer risks due to the ingestion exposures to multiple HMMs of concern. The *TCR* can be evaluated through Equation (9), and the risk value levels are shown in Table 5.

(9) TCR=∑CR

### 2.4. MCS and SA

The sensitivity analysis (SA) approach was used to establish the impact of changing the values of the input variables on the *CR* estimate under a set of assumptions [50]. The SA was implemented using Oracle Crystal Ball^®^ version 11.1.34,190, (Redwood, CA, USA) utilizing the MCS approach with 10,000 iterations in Excel software version 16.0.5332.1000 (Redmond, WA, USA).

### 2.5. Statistical Analysis

Using Excel software, descriptive statistics linked to the physicochemical parameters and HMM concentrations in the DW were evaluated to calculate the mean, standard deviation (SD), and coefficient of variance (CV). The CV was utilized to evaluate the dataset’s variability as follows: CV ≤ 15%, low; 15% < CV ≤ 35%, intermediate; and CV ≥ 35%, high [51,52,53]. A Pearson rank-order correlation analysis coupled with correlation matrix plots was also implemented utilizing IBM SPSS Statistics Version 26.0 and R Studio. Data on the metals concentrations in the domestic water were subjected to a hierarchical cluster analysis (CA) to find homogeneous groups. A dendrogram was likewise produced to analyze the cohesion of the clusters produced, in which relationships among components may easily be identified [54,55].

### 2.6. Spatial Concentration Mapping of Risk Indices Using Machine Learning Geostatistical Interpolation (MLGI) Method

A hybrid neuro-particle swarm optimization (NN-PSO) technique coupled with the empirical Bayesian kriging (EBK) method was utilized to generate spatial maps of the risk indices of domestic water samples from the risk indices calculated at each sampling location [21].

## 3. Results

### 3.1. HMM Concentration in Water Samples

Table 6 shows the average mean, SD, and CV of the DW’s physicochemical parameters and HMM concentrations. These were assessed with WQ standards for drinking water regulated by WHO [1] and PNSDW [56]. The mean pH and TDS of all water samples, except for the mean TDS of TW, were below or within the standards. High TDS may affect the taste and palatability of drinking water [57]. The CV for the TDS and EC of all water samples were higher than 35%, indicating a greater dispersion around the mean and a relatively high variability in the data sets [51].

**Figure 3:**
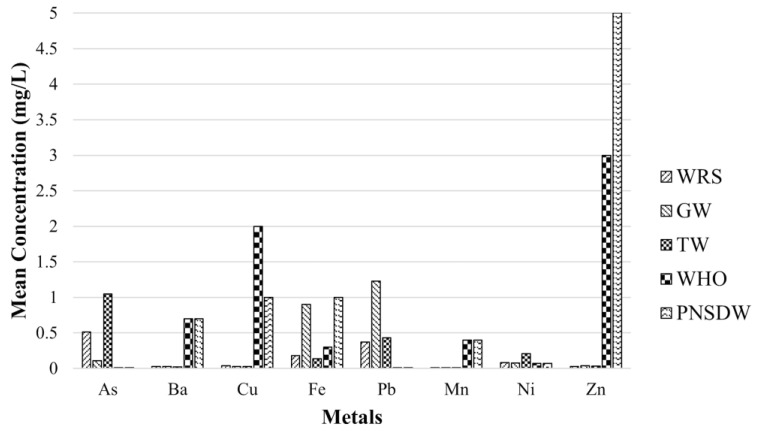
Average HMM concentrations in all DW samples.

The measured HMM concentrations in the DW shown in Figure 3 varied between 0 and 19.0 mg/L for WRS, 0 and 13.4 mg/L for GW, and 0 and 9.31 mg/L for TW. The mean concentrations of As, Pb, and Ni in WRS, GW, and TW were higher than the acceptable limits of the WHO and PNSDW standards. These elevated concentrations of HMM were attributed to the existing two abandoned open mine pits that are located at a higher elevation and sit on permeable volcanic and sedimentary rocks [14,24] that allow groundwater to pass through.

The observed HMM concentrations trend, shown in Table 7, for WRS, GW and TW were As > Pb > Fe > Ni > Cu > Zn > Ba > Mn, Pb > Fe > As > Ni > Zn > Ba > Cu > Mn, and As > Pb > Ni >Fe > Zn > Cu > Ba > Mn, respectively. It was recorded that As had the highest concentration detected in both the WRS and TW, while Mn had the lowest concentration observed in all DW sources.

Continuous subsurface flow of HMM into inland waters contributes to the increased metal concentration in water resources. In addition, the weathering of rocks that leached HMMs may contribute to the concentration of these HMM. 

### 3.2. MPI and NPI Results

*MPI* and *NPI* were broadly utilized to evaluate the total HMM contamination in water resources [4]. The *MPI* calculated in all DW sampling locations was observed to have a high degree of pollution. The average *MPI* value for the TW samples was 37.5 times more than the minimum *MPI* value. This is classified as having a high degree of pollution, while the average *MPI* values for the GW and WRS samples were 33.6 times and 22.3 times greater, respectively. Moreover, the *NPI* values observed in the DW samples were 8.4 times to 13.6 times higher than the minimum *NPI* value. These *NPI* values are categorized as heavily polluted.

### 3.3. Human HR Assessment

**Figure 4:**
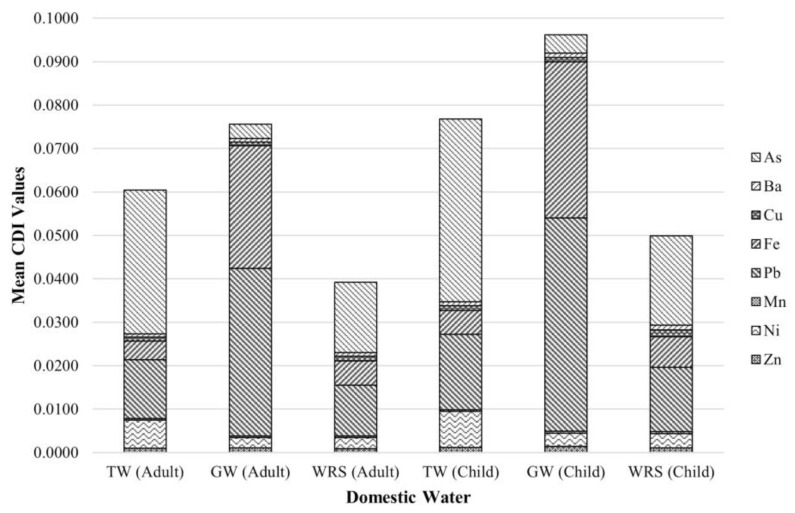
Mean *CDI* of metals in water from WRS, groundwater, and tap water.

Table 8 presents the mean *CDI* of metals through the oral route from DW. The *CDI* values calculated for adults ranged from 0.0003 to 0.0386 for all DW sources. At the same time, the *CDI* values for children were observed to range from 0.0004 to 0.0491. The highest mean *CDI*, illustrated in Figure 4, for adults and children was observed for As for TW and WRS and Pb for GW. The smallest mean *CDI* for adults was observed for Mn for all DW samples. The study of de Jesus et al. (2021) in Marinduque also revealed high concentrations of Pb in the GW samples [58].

The *HQ* indices of metals through water intake in the study area are elaborated in Table 9. The average *HQ* indices of As for adults and children in TW and WRS were highest, while the mean *HQ* index value for Pb was the highest for GW samples both for adults and children. The *HQ* index trend of HMM for adult and children in TW and WRS were observed to be As > Pb > Ni > Cu > Fe > Ba > Zn > Mn, while the *HQ* index in GW for adult is Pb > As > Ni > Fe > Cu > Ba > Mn > Zn. The *HQ* index trend of GW for children was Pb > As > Ni > Fe > Cu > Ba > Zn > Mn. Furthermore, the *HQ* indices of As and Pb for all water sources were greater than 1, indicating potential health risks to the human population. It must be emphasized that exposure to HMM may also come from various sources and through other pathways of exposure [59].

**Figure 5:**
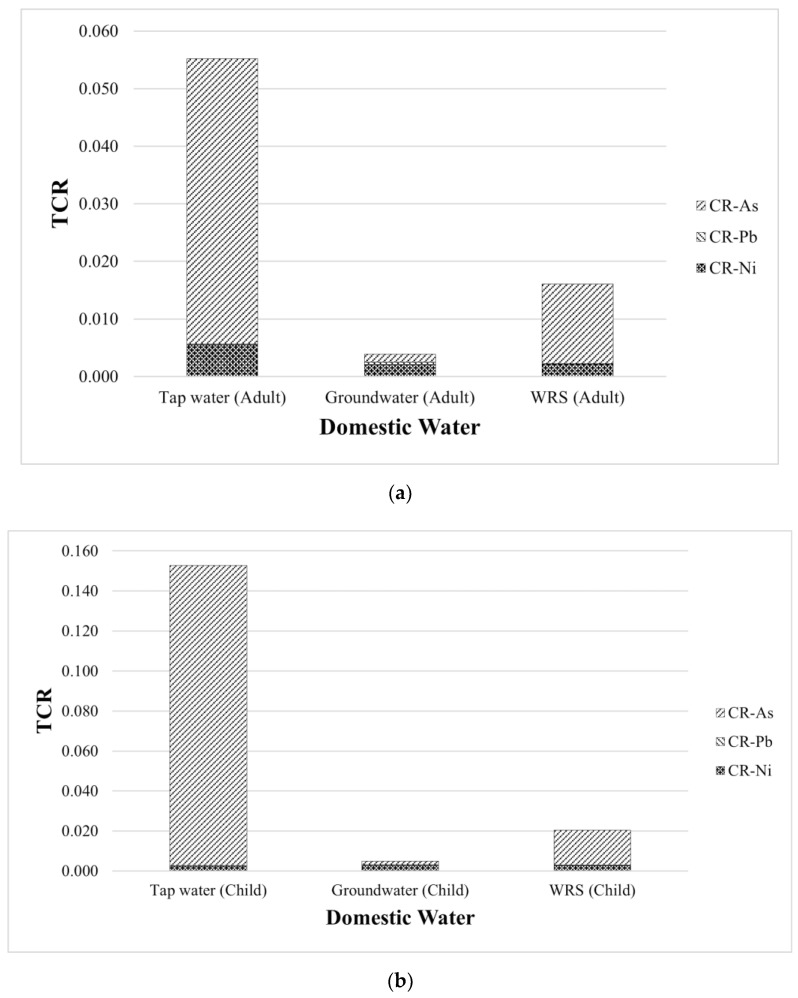
Distribution of *TCR* in domestic water (DW) sources for (**a**) adults and (**b**) children.

The *TCR* to residents through water intake from TW, GW, and WRS is summarized in Table 10. Among the studied target contaminants, only As, Pb, and Ni are categorized as carcinogenic metals by the International Agency for Research on Cancer (IARC) [60,61]. The adult carcinogenic risk for As ranged from 1.41 × 10^−3^ to 4.96 × 10^−2^; Pb from 8.92 × 10^−5^ to 4.16 × 10^−4^; and Ni from 2.07 × 10^−3^ to 5.50 × 10^−3^. The child carcinogenic risk for As ranged from 1.80 × 10^−3^ to 1.50 × 10^−1^; Pb from 1.14 × 10^−4^ to 5.29 × 10^−4^; and Ni from 2.35 × 10^−3^ to 2.76 × 10^−3^. The mean *TCR* was 2.51 × 10^−2^ and 5.95 × 10^−2^ for adults and children, respectively. All these risks were greater than the threshold value proposed by USEPA, which is 1 × 10^−4^ [62,63]. Having recorded these values, certain interventions and control measures are required to reduce the level of concentrations of HMM in the province and limit the population’s exposure. Adequate remediation and prompt onsite treatment to safeguard people’s health are necessary [64]. The *TCR* of the carcinogenic metals in all water sources were seen in the order As > Ni > Pb, and the *TCR* was in the order TW > WRS > GW both for adult and children, as shown in Figure 5.

### 3.4. Monte Carlo Simulation and Sensitivity Analysis

**Figure 6:**
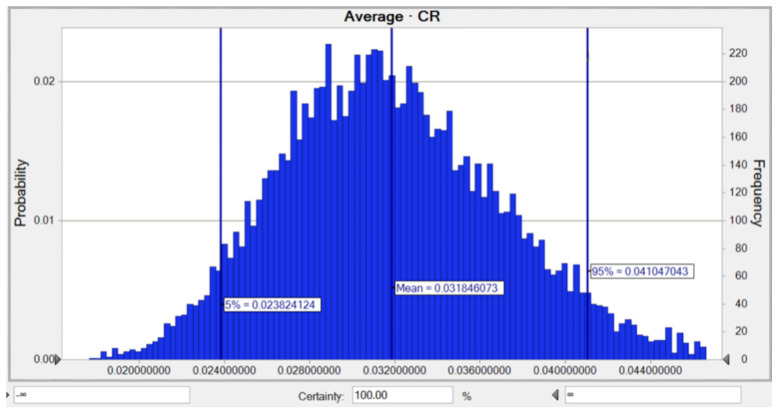
Predicted probability of *TCR* (adults) for As in water.

The carcinogenic risk (adults and children) related to As, Pb, and Ni in all residential water sources in Marinduque, Philippines was evaluated using the Monte Carlo method. The likelihood of lifetime cancer risk (adults) for As in all water samples is shown in Figure 6. The mean *TCR* for As was 3.18 × 10^−2^, while the risks of 5% and 95% were as high as 2.38 × 10^−2^ and 4.10 × 10^−2^, respectively. This risk is very high compared to the indicated maximum acceptable risk of 1.00 × 10^−4^ for As.

**Figure A1:**
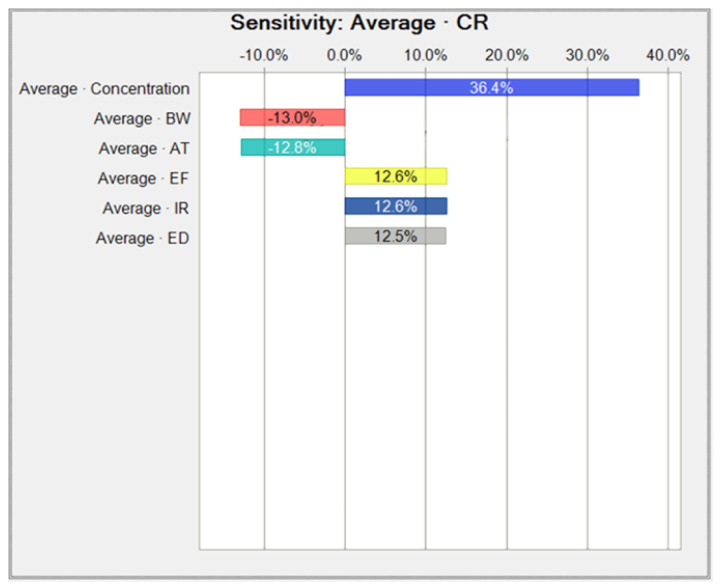
SA of LTCR model (adults) for As in water.

The factors included in the lifetime carcinogenic risk (LTCR) estimate was then determined using a sensitivity analysis (SA). The As in water demonstrated that the two components involving HMM content and body weight (BW) had the most significant effect on the LTCR values. Compared to other factors, an As concentration of 36.4 percent and a BW concentration of 13.0 percent had the highest positive and negative impacts on the LTCR calculation (Figure A1).

**Figure 7:**
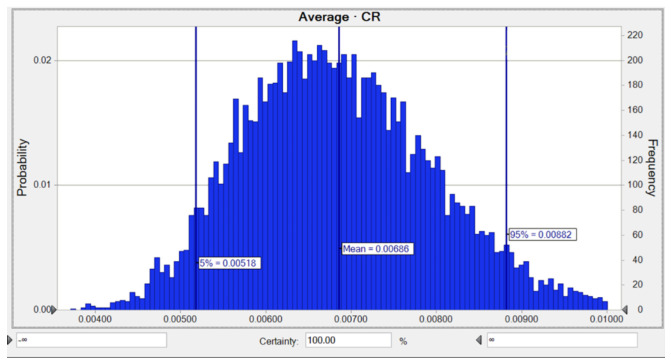
Predicted probability of *TCR* (Adult) for Ni in water.

**Figure A2:**
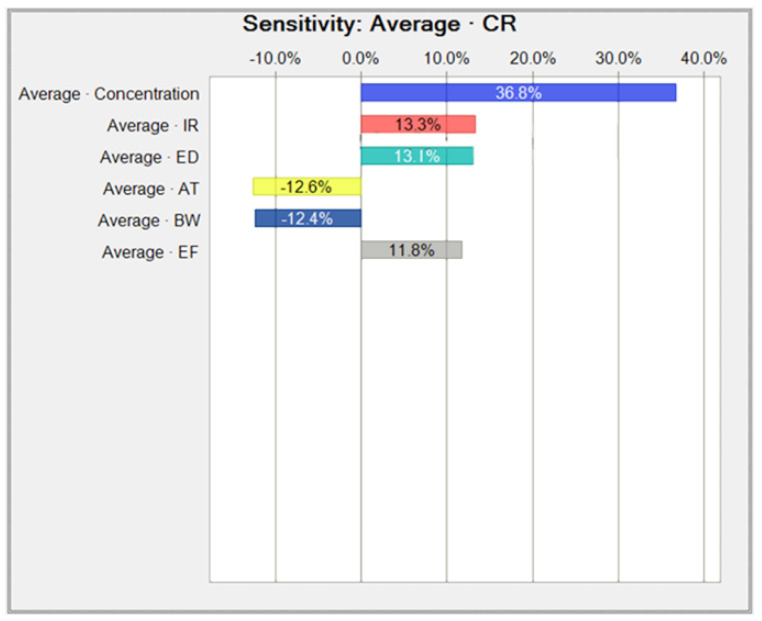
SA of LTCR model (adults) for Ni in water.

The average likelihood of the LTCR for Ni was 6.86 × 10^−3^, while the risks of 5% and 95% were equal to 5.18 × 10^−3^ and 8.82 × 10^−3^, respectively (Figure 7). The SA for the LTCR estimate regarding Ni indicated that the two parameters including a Ni concentration of 36.8% and an AT of −12.6%, respectively, had the highest positive and negative influences on the carcinogenic hazard value, as shown in Figure A2.

**Figure 8:**
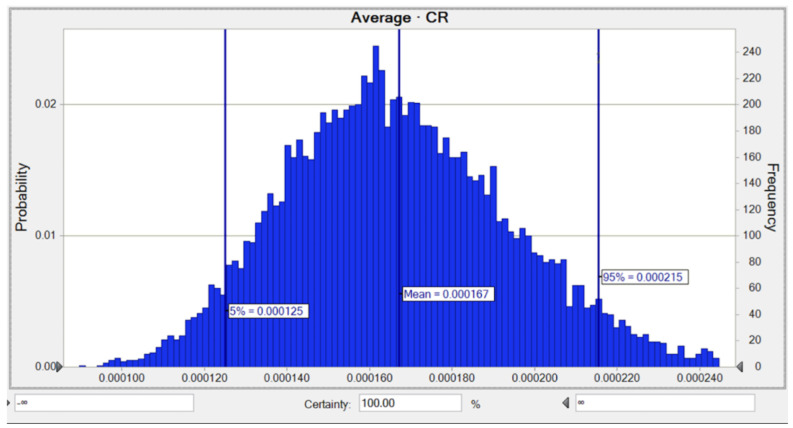
Predicted probability of *TCR* (Adult) for Pb in water.

**Figure A3:**
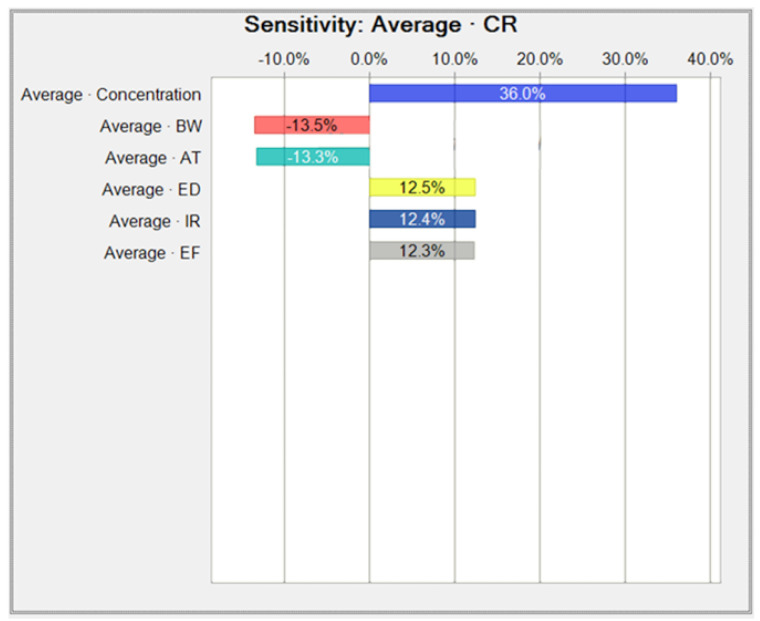
SA of LTCR model (adults) for Pb in water.

Moreover, the average likelihood of lifetime CR for Pb was 1.67 × 10^−4^, while the risks of 5% and 95% were equal to 1.25 × 10^−4^ and 2.15 × 10^−4^, respectively, as shown in Figure 8. The SA for the LTCR computation concerning Pb demonstrated that the two parameters consisting of a Pb concentration of 36.0% and a BW of −13.5% had the highest positive and negative impacts on the carcinogenic hazard value as shown in Figure A3.

**Figure 9:**
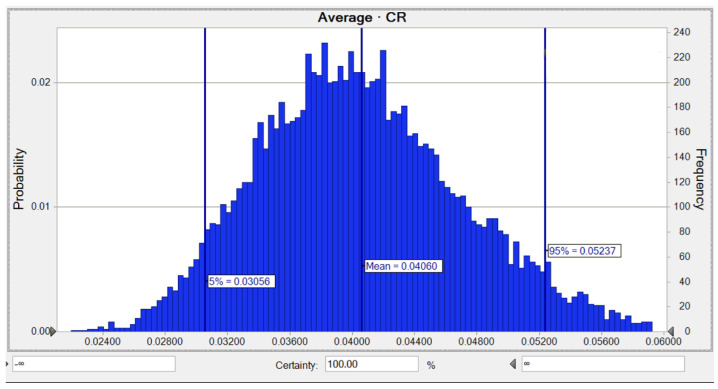
Predicted probability of *TCR* (children) for As in water.

**Figure A4:**
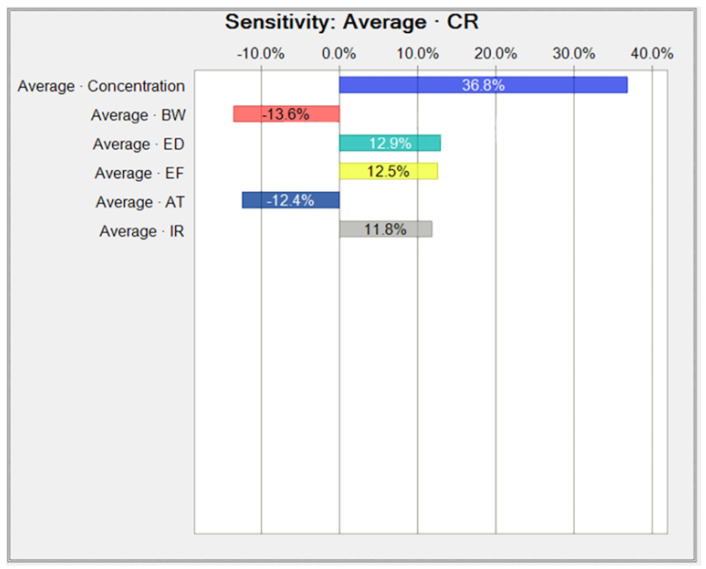
SA of LTCR model (children) for As in water.

For children, the average likelihood of LTCR of As was observed to be 4.06 × 10^−2^, while the risks of 5% and 95% were equivalent to 3.06 × 10^−2^ and 5.24 × 10^−2^, respectively, as shown in Figure 9. The SA for the LTCR (children) computation on As demonstrated that the two parameters comprising an As concentration of 36.8% and a BW of −13.6% had the highest positive and negative impacts on carcinogenic hazard computation as shown in Figure A4.

**Figure 10:**
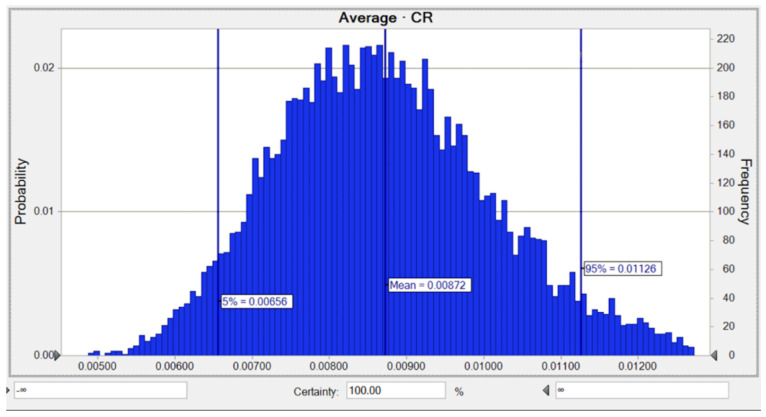
Predicted probability of *TCR* (children) for Ni in water.

**Figure A5:**
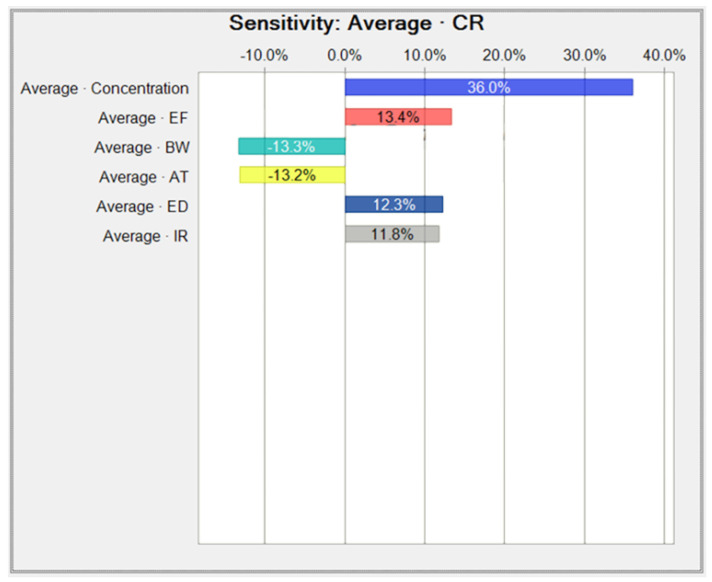
SA of LTCR model (children) for Ni in water.

The average likelihood of LTCR of Ni for children was observed to be 8.72 × 10^−3^, while the risks of 5% and 95% were equivalent to 6.56 × 10^−3^ and 1.13 × 10^−2^, respectively as shown in Figure 10. The SA for the LTCR (children) computation involving Ni explained that the two parameters containing a Ni concentration of 36.0% and a BW of −13.3% had the highest positive and negative impacts on the carcinogenic hazard calculation, as shown in Figure A5.

**Figure 11:**
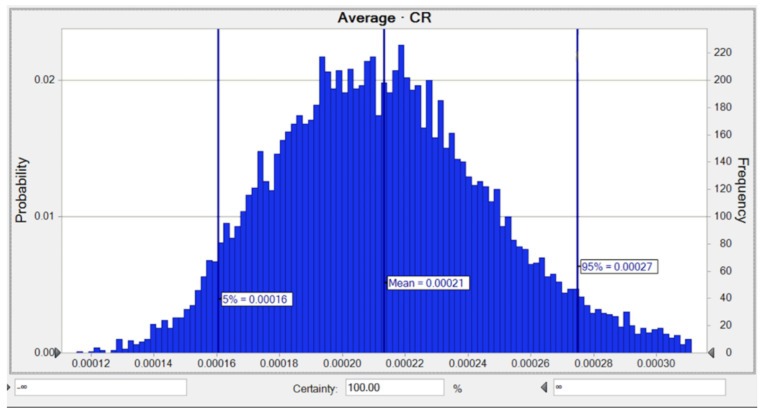
Predicted probability of *TCR* (children) for Pb in water.

**Figure A6:**
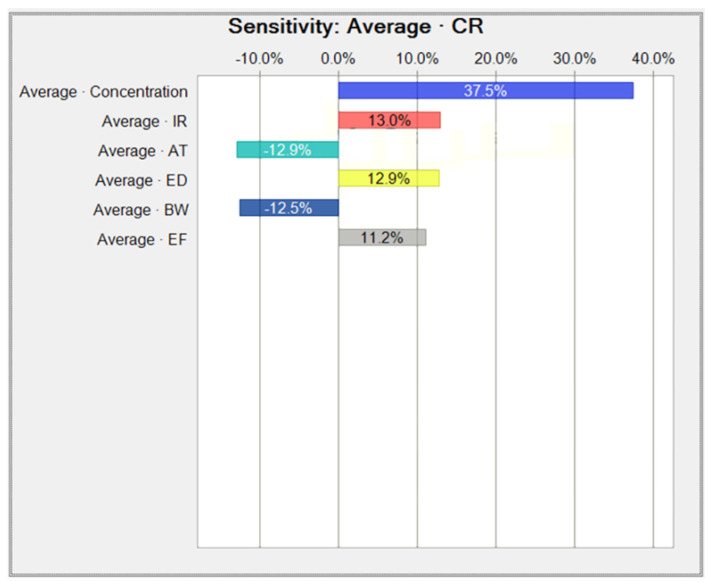
SA of LTCR model (children) for Pb in water.

Furthermore, the LTCR of Pb for children was observed to be 2.10 × 10^−4^, while the risks of 5% and 95% were equivalent to 1.60 × 10^−4^ and 2.70 × 10^−4^, respectively as shown in Figure 11. The SA for the LTCR (children) computation involving Pb revealed that the two parameters consisting of a Pb concentration of 37.5% and an AT of −12.9% had the highest positive and negative impacts on the carcinogenic hazard calculation, as shown in Figure A6.

### 3.5. Relationship of WQ Parameters in the Domestic Water Samples

**Figure 12:**
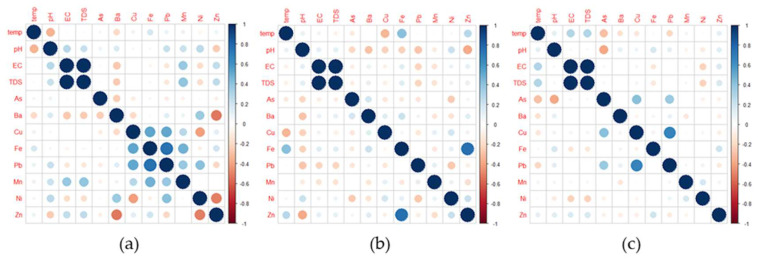
Correlation matrix plots of WQ parameters obtained for (**a**) TW samples, (**b**) GW samples, and (**c**) WRS samples.

The relationships between the selected WQ parameters, especially metals, in the water provided interesting information on their potential sources and pathways. Figure 12a presents the correlation plot for the WQ parameters obtained from the TW samples. It can be seen that there are significant positive correlations between EC and TDS as well as Pb and Cu. For the GW samples, the correlation matrix plot of each WQ parameter is presented in Figure 12b. It is observed that significant positive correlations exist between EC and TDS and Fe and Zn. Moreover, the correlation matrix plot of WQ parameters obtained for WRS is exhibited in Figure 12c. It is observed that significant positive correlations exist between EC and TDS, Fe and Cu, Pb and Cu, and Pb and Fe.

**Figure 13:**
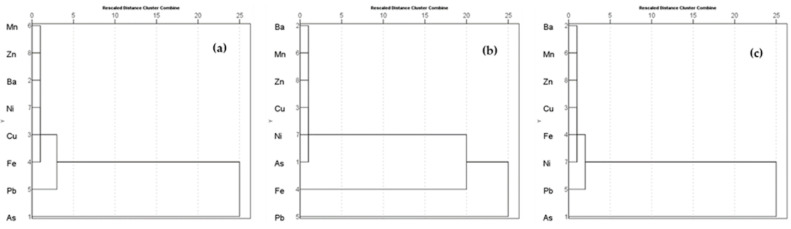
Cluster analysis dendrogram of HMMs in (**a**) WRS, (**b**) GW, and (**c**) TW.

The observed relationships of metals in all water sources were further supported by a hierarchical cluster analysis (CA) dendrogram. The primary clusters found in WRS were (1) Mn-Zn-Ba-Ni-Cu-Fe, (2) Pb, and (3) As (Figure 13a). The primary clusters for GW were (1) Ba-Mn-Zn-Cu-Ni-As, (2) Fe, and (3) Pb, as shown in Figure 13b. Lastly, the primary clusters found in TW were (1) Ba-Mn-Zn-Cu-Fe-Ni, (2) Pb, and (3) As (Figure 13c).

### 3.6. Pollution and Health Risk Mapping Using the MLGI Method

**Figure 14:**
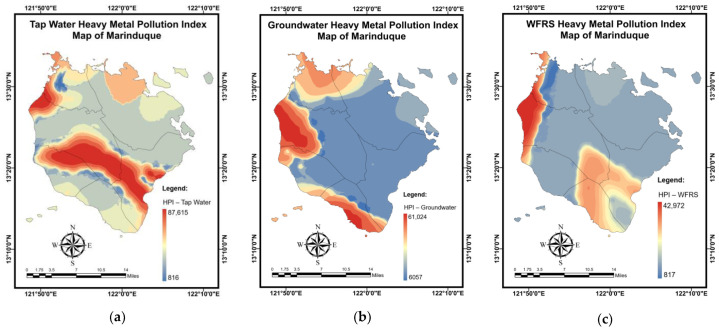
Spatial maps of *MPI* developed using MLGI approach for (**a**) TW samples, (**b**) GW samples, and (**c**) WRS samples.

**Figure A7:**
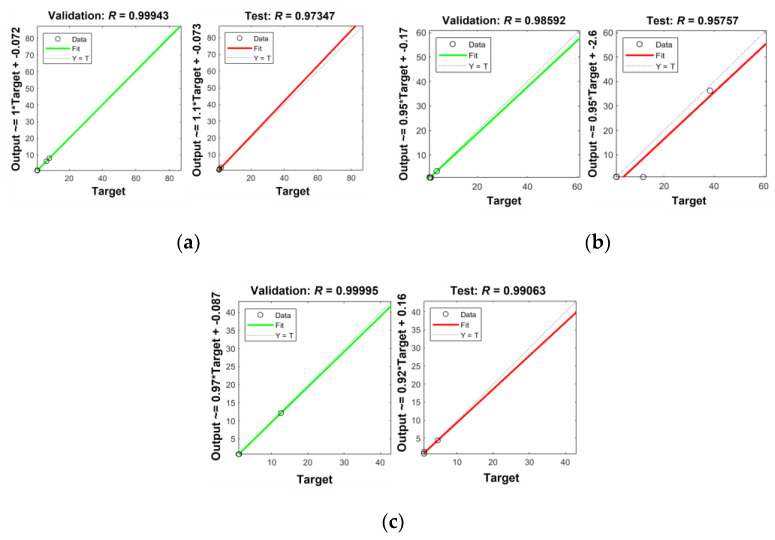
Correlation plots for NN-PSO simulations of *MPI* for (**a**) TW, (**b**) GW, and (**c**) WRS.

The results of the simulation for the *MPI* mapping of the DW samples using the NN-PSO technique integrated with the EBK method are presented in Table 11. The validation and testing plots of the *MPI* for the DW samples are shown in Figure A7 in Appendix B. As shown in Figure 14a, the highest *MPI* for TW was observed in Brgy. Market Site, Municipality of Mogpog. The highest *MPI* was observed in Brgy. Bicas-Bicas, Municipality of Buenavista for the GW samples (Figure 14b). Moreover, it was observed that the highest *MPI* for WRS was in Brgy. Janagdong, Municipality of Mogpog as shown in Figure 14c.

**Figure 15:**
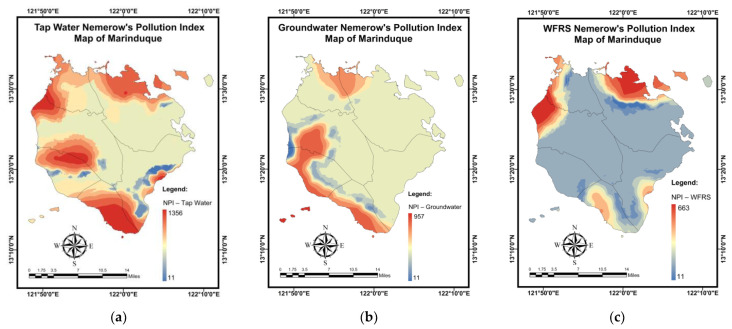
Spatial maps of *NPI* developed using MLGI approach for (**a**) TW samples, (**b**) GW samples, and (**c**) WRS samples.

**Figure A8:**
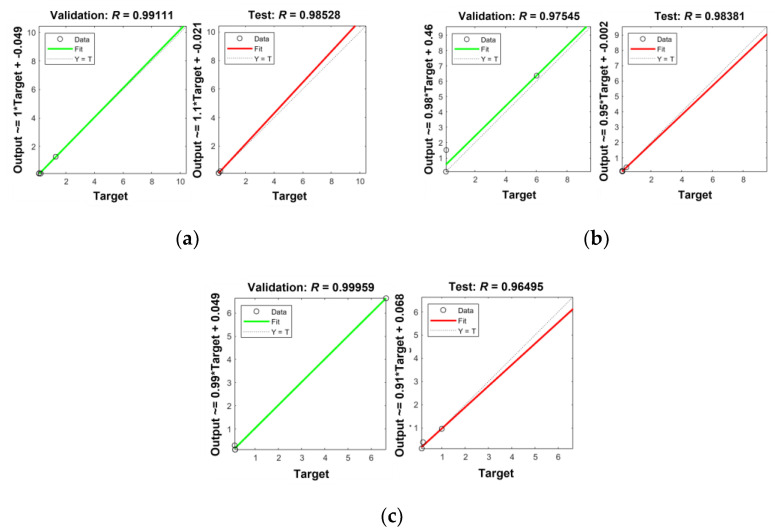
Correlation plots for NN-PSO simulations of *NPI* for (**a**) TW, (**b**) GW, and (**c**) WRS.

Table 12 shows the simulation results for the *NPI* mapping of DW samples using the NN-PSO approach combined with the EBK method. The validation and testing plots for the *NPI* maps are illustrated in Figure A8 in Appendix B. As seen in Figure 15a, Brgy. Buangan, Torrijos reported the highest *NPI* for TW. The highest *NPI* values for GW were found in Brgy. Bicas-Bicas, Municipality of Buenavista as shown in Figure 15b. Additionally, the highest *NPI* for WRS was recorded in Brgy. Janagdong, Mogpog Municipality, as shown in Figure 15c.

**Figure 16:**
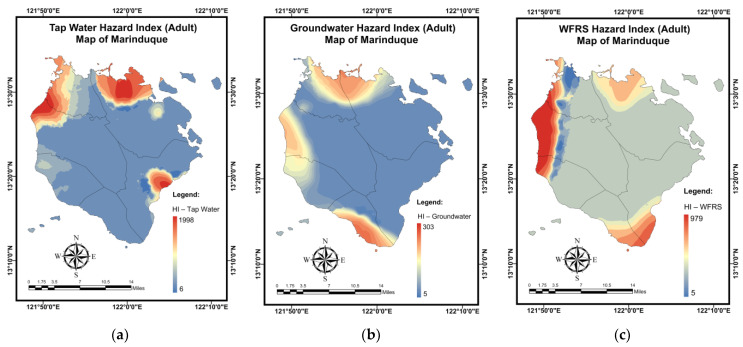
Spatial maps of HI (adults) developed using MLGI approach for (**a**) TW samples, (**b**) GW samples, and (**c**) WRS samples.

**Figure A9:**
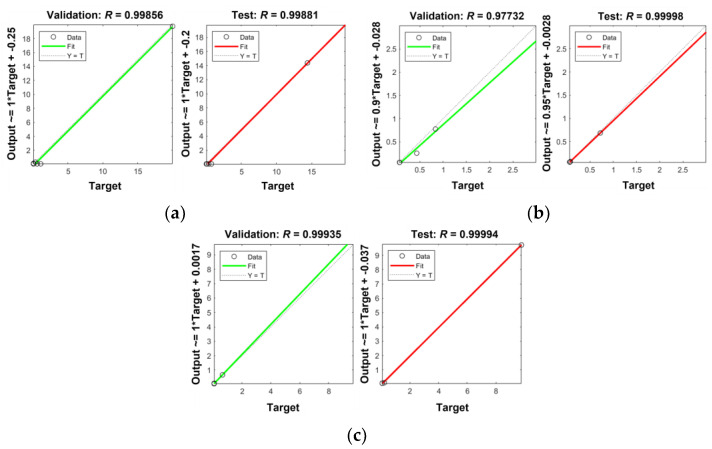
Correlation plots for NN-PSO simulations of HI (adults) for (**a**) TW, (**b**) GW, and (**c**) WRS.

The findings of a simulation for the *HI* (adults) mapping of domestic water samples using the NN-PSO approach with the EBK technique are shown in Table 13. The validation and testing plots for the *HI* (adults) maps are illustrated in Figure A9 in Appendix B. As shown in Figure 16a, Brgy. Buangan, Torrijos reported the highest *HI* (adults) for tap water. As seen in Figure 16b, the highest *HI* (adults) values for GW were observed in Brgy. Bicas-Bicas, Municipality of Buenavista. Additionally, the highest *HI* (adults) for WRS was recorded in Brgy. Janagdong, Mogpog Municipality as shown in Figure 16c.

**Figure 17:**
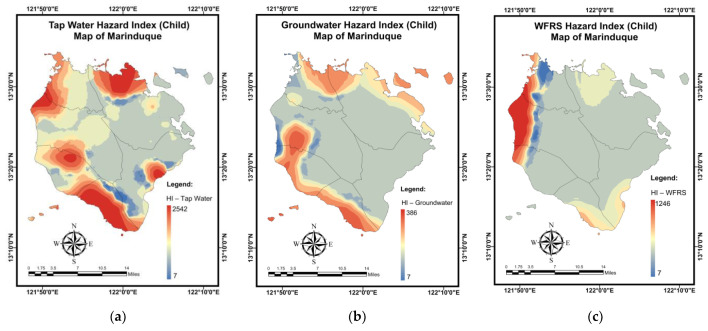
Spatial maps of *HI* (children) developed using MLGI approach for (**a**) TW samples, (**b**) GW samples, and (**c**) WRS samples.

**Figure A10:**
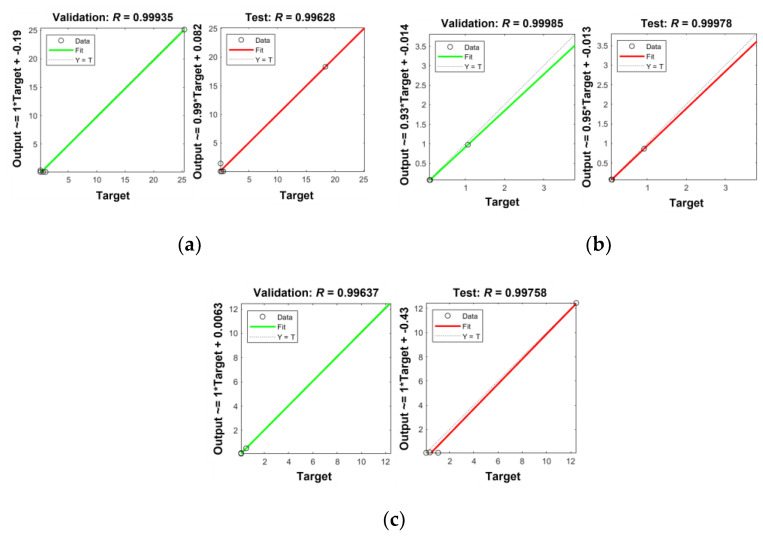
Correlation plots for NN-PSO simulations of *HI* (children) for (**a**) TW, (**b**) GW, and (**c**) WRS.

Table 14 summarizes the results of a simulation for the *HI* (children) mapping of residential water samples using the NN-PSO methodology integrated with the EBK method. Figure A10 in Appendix B illustrates the validation and testing plots for the *HI* (children) maps. Brgy. Buangan, Torrijos recorded the highest *HI* (children) for tap water, as seen in Figure 17a. As seen in Figure 17b, the highest *HI* (children) values for GW were found in Brgy. Bicas-Bicas, Buenavista Municipality. Additionally, as shown in Figure 17c, the highest *HI* (children) for WRS was obtained in Brgy. Janagdong, Mogpog Municipality.

**Figure 18:**
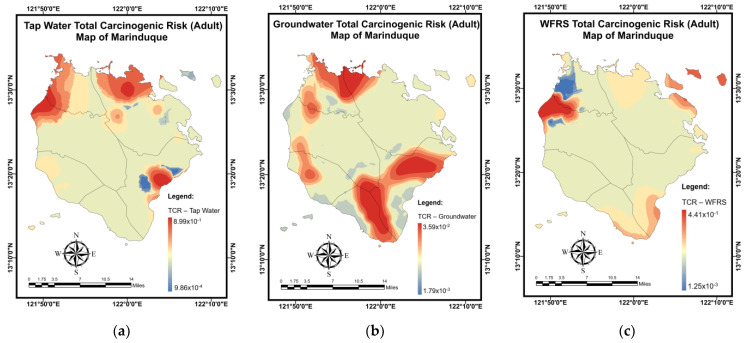
Spatial maps of *TCR* (adult) developed using MLGI approach for (**a**) TW samples, (**b**) GW samples, and (**c**) WRS samples.

**Figure A11:**
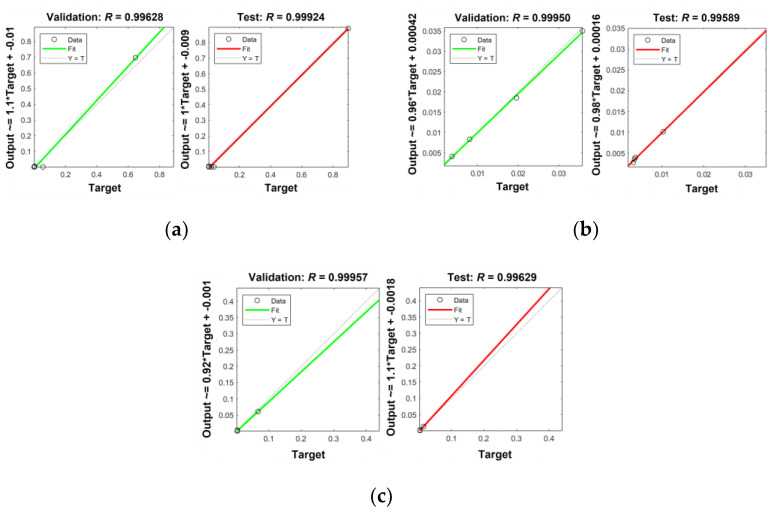
Correlation plots for NN-PSO simulations of TCR (adults) for (**a**) TW, (**b**) GW, and (**c**) WRS.

The findings of a simulation for the *TCR* (adults) mapping of domestic water samples using the NN-PSO approach with the EBK method are shown in Table 15. The validation and testing plots for the *TCR* (adults) maps are illustrated in Figure A11 in Appendix B. As shown in Figure 18a, Brgy. Buangan, Torrijos reported the highest *TCR* (adults) for TW. As illustrated in Figure 18b, the highest *TCR* (adults) levels for GW were found in Brgy. Bagacay, Buenavista. Additionally, the highest *TCR* (adults) for WRS was recorded in Brgy. Janagdong, Mogpog Municipality as shown in Figure 18c.

**Figure 19:**
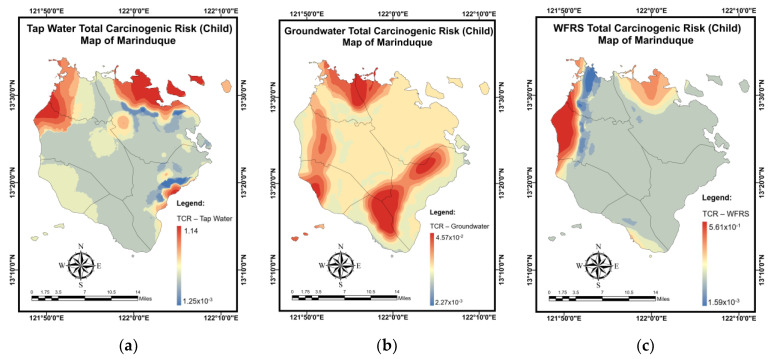
Spatial maps of *TCR* (child) developed using MLGI approach for (**a**) TW samples, (**b**) GW samples, and (**c**) WRS samples.

**Figure A12:**
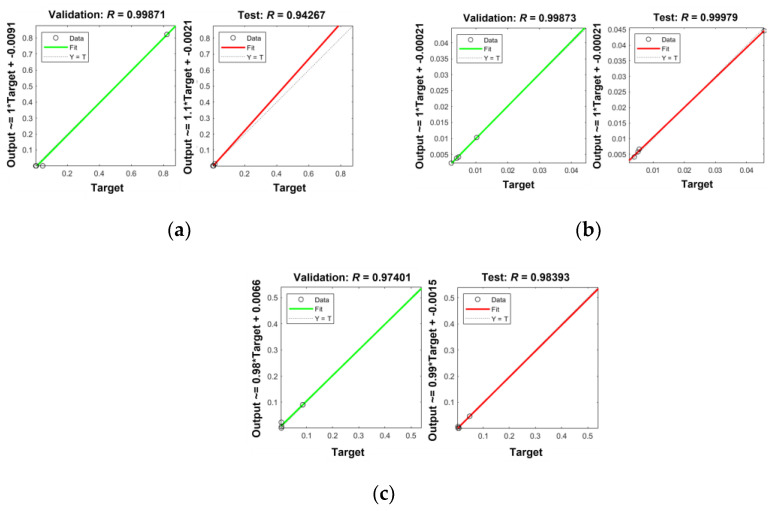
Correlation plots for NN-PSO simulations of TCR (children) for (**a**) TW, (**b**) GW, and (**c**) WRS.

Table 16 summarizes the simulation results for the *TCR* (children) mapping of domestic water samples using the NN-PSO methodology integrated with the EBK method. Figure A12 in Appendix B illustrates the validation and testing plots for the *TCR* (children) maps. Brgy. Buangan, Torrijos reported the highest *TCR* (children) for TW, as seen in Figure 19a. As illustrated in Figure 19b, the highest *TCR* (children) levels for GW were found in Brgy. Bagacay, Buenavista. Additionally, the highest *TCR* (children) for WRS was recorded in Brgy. Janagdong, Mogpog Municipality as shown in Figure 19c.

**Figure 20:**
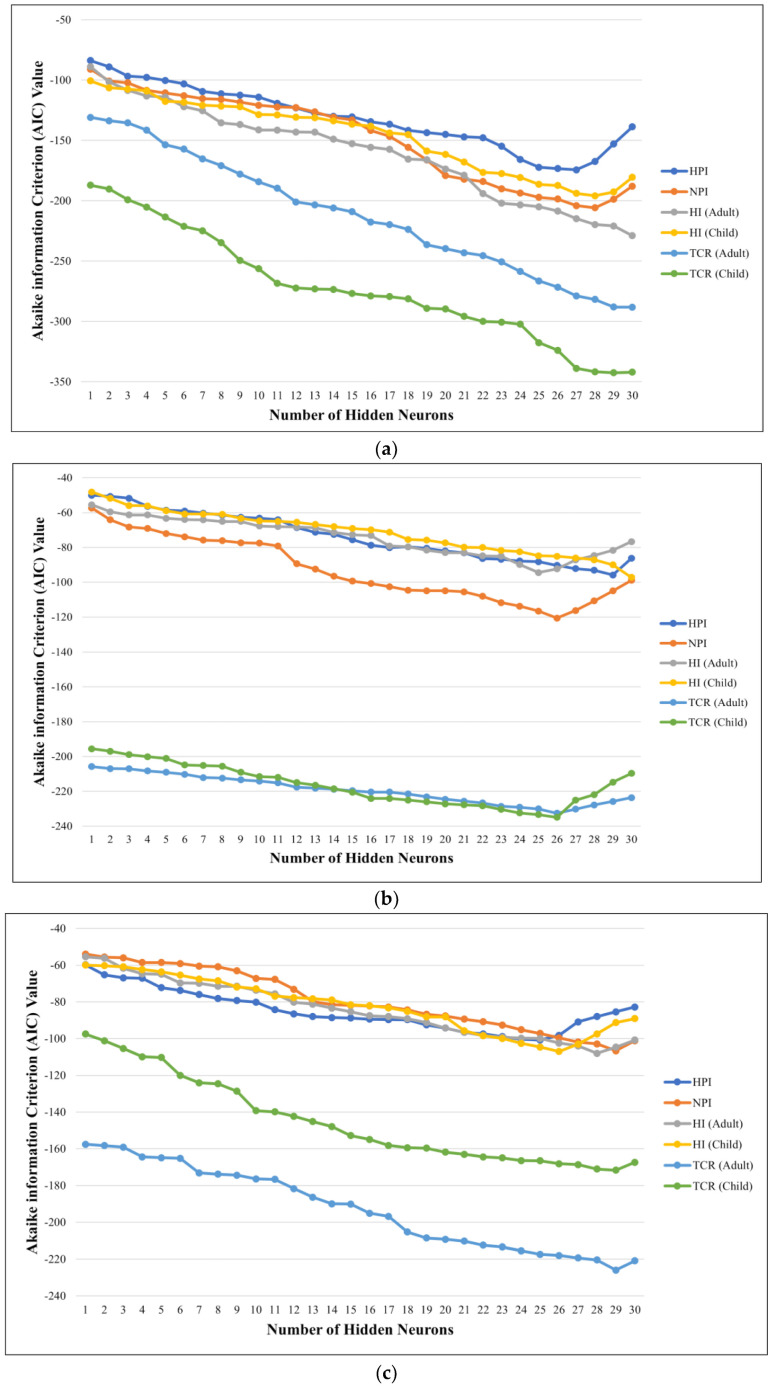
Akaike information criterion (AIC) values for pollution and risk indices in (**a**) TW, (**b**) GW, and (**c**) WRS.

The association between the number of neurons simulated from 1 to 30 and the AIC (Akaike information criterion) values calculated for the pollution and risk indices (*MPI*, *NPI*, *HI* (adults), *HI* (children), *TCR* (adults), and *TCR* (children)) in TW, GW, and WRS are exhibited in Figure 20a, Figure 20b, and Figure 20c, respectively. These figures provide the AIC values for all NN-PSO models used in this research for each hidden neuron simulated. The best models for the tap water models had 27, 28, 30, 28, 30, and 29 hidden neurons for *MPI*, *NPI*, *HI* (adults), *HI* (children), *TCR* (adults), and *TCR* (children), respectively. For the groundwater, the best models were observed with 29, 26, 25, 30, 26, and 26 hidden neurons for *MPI*, *NPI*, *HI* Adults, *HI* (children), *TCR* (adults), and *TCR* (children), respectively. Moreover, for the WRS, the best models observed were found with 25, 29, 28, 26, 29, and 29 hidden neurons for *MPI*, *NPI*, *HI* (adults), *HI* (children), *TCR* (adults), and *TCR* (children), respectively.

## 4. Discussion

Identifying the parameters influencing the GW chemistry is vital for the sustainability of water resources [65]. In this study, it was observed that the mean concentrations of As, Pb, and Ni in domestic water resources in the province of Marinduque were above the limit set by the WHO. The relationship of water quality parameters was likewise determined, and it was recorded that there was a substantial positive correlation between EC and TDS in tap water samples, which agrees with the results of the study by Qureshi et al. [66]. Moreover, a high positive correlation was also seen between Pb and Cu in TW and Pb and Fe in WRS, which is similar to the findings of Varghese and Jaya [67]. For WRS, Fe and Cu had a significant positive correlation that agreed with the results of Kuisi and Abdel-Fattah [68]. A positive correlation among these metals suggests a possible shared origin [69].

The *MPI* and *NPI* were broadly utilized in the evaluation of the total HMM contamination in GW. The *MPI* covers the weight of different HMMs in the computation of the overall quality of drinking water [4,70]. In the present study, HMM including As, Ba, Cu, Fe, Pb, Mn, Ni, and Zn were considered. The findings showed that all sampling locations recorded *MPI* values classified as having a high degree of pollution. Similarly, all sampling points were heavily polluted based on the calculated *NPI* values. As, Pb, and Ni in all water sources exceeded both the water quality standards of WHO and PNSDW. Similar findings were observed by Agarin et al. [14], who investigated the concentration of metals in the surface and groundwater of the island province in 2021.

The total carcinogenic risk computed through the ingestion route exceeded the maximum threshold level of 1.00 × 10^−4^ [71] for all DW samples. The results indicated that children were more vulnerable to *CR* than adults, as shown by the observation that the *TCR* values for children were all greater than the *TCR* values calculated for adults, consistent with the results of Pervez et al. [35]. Therefore, it is essential to determine the As levels because exposure may cause either acute or chronic poisoning. Acute As toxicity is often characterized by nausea, vomiting, abdominal discomfort, and severe watery diarrhea [72]. Chronic arsenic effects may arise due to prolonged exposure to lower arsenic levels, although latent toxicity, such as cancer, can persist even after exposure has ended. Chronic arsenic toxicity may gradually develop, making it more difficult to identify. Skin manifestations and peripheral neurologic complaints are often more apparent than gastrointestinal symptoms, with chronic exposure and concerns also centering on an increased future risk of cancer [73]. Early skin indicators include hyperpigmentation or hypopigmentation of the skin. Hyperkeratosis and scaling, especially on the palms and soles, highly indicate arsenic exposure [74]. Carcinomas of the skin and Bowen’s disease are connected with latent arsenic toxicity. Additionally, a peripheral vascular condition known as a Blackfoot disease accompanying gangrene has been related to chronic arsenic exposure [75,76]. Numerous research and case reports have also shown a link between arsenic exposure and cancer. Arsenic exposure has been linked to skin [77], lung [78], liver [79], kidney [80], bladder [81], and prostate cancers [82].

Similar to As toxicity, Pb toxicity can have acute or chronic effects. Acute Pb toxicity symptoms associated with Pb include dullness [83], restlessness [84], irritability [85], short attention span [86], headaches [87], muscular tremor [88], abdominal pain [89], renal damage [90], hallucinations [91], memory loss [92], and encephalopathy [93]. Moreover, signs of chronic Pb toxicity include tiredness [94], sleeplessness [95], headaches [96], joint pain [97], and gastrointestinal symptoms [89]. Furthermore, Pb exposure raises the risk of lung, stomach, and bladder cancer [98]. Additionally, Ni toxicity causes headaches [99], gastrointestinal manifestations [100], cardiovascular diseases, and cancer [101]. In 2020, the provincial health office of the island province reported cancer deaths mainly from the municipalities of Mogpog and Sta. Cruz. Although there is no report on the pathogenesis of these cancer cases, environmental quality influences the development and severity of diseases such as cancer.

The different pollution and health risk indices were mapped using the MLGI approach, which combines NN-PSO with EBK [21]. The governing MLGI models were found to be established from the AIC measure values that were the lowest among the observable hidden neurons. As the AIC value approached its minimal value, it was revealed that increasing the quantity of hidden neurons resulted in an increased AIC value. This indicated that the network had reached a state of generalization [58].

The concentrations of HMM do not necessarily reflect the actual pollution state of a water resource since it only assesses each heavy metal separately and with equal severity in terms of its biological effects. The application of pollution and health risk indices could provide a more conclusive assessment of the pollution and health risk status of a region since it compares the concentrations of each heavy metal to their acceptable levels [102]. It also indicates the total amount of pollution a region is facing. These indices were applied to an island province that was hit by two mine tailing disasters and it could be adopted in other locations across the world that experienced similar mining disasters.

The utilization of pollution and health risk indices is critical for ensuring the quality of water resources consumed and used by residents. These indices can be employed to determine the current risk to which the community may be exposed and recommend potential mitigation measures to limit the level of exposure of community members [103]. The incorporation of the MLGI approach enables the creation of spatial maps of various indices to provide remediation strategies and decision-making processes.

## 5. Conclusions

This study examined the spatial variability of the *MPI*, the *NPI*, the *HI* and the *TCR* (for children and adults) by HMM such as As, Ba, Cu, Fe, Pb, Mn, Ni, and Zn in an island province’s DW sources, i.e., TW, GW, and WRS. Moreover, it highlighted the use of portable devices for in situ detection of HMMs in water, providing accurate and real-time results. It was recorded that As, Pb, and Ni concentrations were much higher than the WHO’s permissible levels. The correlation analysis and dendrogram revealed that the link between the HMMs detected indicated a common origin. The computed *MPI* values in all sampling sites for all domestic water samples were characterized as highly polluted. Additionally, the *NPI* values estimated at all sampling stations for all DW samples were categorized as heavily polluted. The *HQ* indices for As and Pb were significantly greater than 1 for all water sources, indicating a potentially significant health risk to the human population. Moreover, As had the highest *CR* observed in the tap water samples and accounted for 89.7% of the total *CR* in TW. Additionally, all sample sites exceeded the USEPA’s suggested maximum threshold level of 1.00 × 10^−4^, which indicates a high carcinogenic risk. The sensitivity analysis results using MCS showed that the most influential variable in the LTCR was the contaminant concentration. Further, reducing the metal concentration reduced the carcinogenic risk. The calculated indices and MLGI-based maps can be utilized as a benchmark for future research by local government units. It would be helpful in the creation and implementation of remediation and mitigation strategies to promote sustainable development in the protection of domestic water resources. Prompt intervention is required, as well as a regular monitoring of the quality of domestic water sources in the island province. This is to reduce the negative health impacts of these elevated HMM to the local population. Priority and special attention shall be given to the HMM that have elevated concentration and exceed the permissible limits by WHO and PNSDW. In addition, a regular monitoring of water resources quality for domestic supply shall be carried out, the development of policies based on these results shall be pursued, and the enforcement of existing policies shall be effectively conducted to reduce adverse health effects to the population of the island province.

## Figures and Tables

**Table 1:** Categorization of WQ using *MPI* [30].

Method of Indexing	Range	Degree of Pollution
Heavy Metal Pollution Index (*MPI*)	<90	Low
	90–180	Medium
	>180	High

**Table 2:** Classification of *NPI* values [34].

*NPI*	Contamination Degree
<1.0	Unpolluted
1.0 ≤ P_N_ < 2.5	Slightly polluted
2.5 ≤ P_N_ < 7.0	Moderately polluted
≥7.0	Heavily polluted

**Table 3:** Parameters included in the calculation of *CDI_in_*.

Parameter	Unit	Oral Values	Investigator(s)
Ingestion rate (IR)			
✓ Adult	Liters/day	2.20	[37]
✓ Child	Liters/day	1.00	[38]
Exposure frequency (*EF*)	Days/year	365	[39]
Exposure duration (*ED*)			
✓ Adult	Year	70	[40]
✓ Child	Year	10	[40]
Body weight (*BW*)			
✓ Adult	Kilograms	70	[37]
✓ Child	Kilograms	25	[38]
Average time (*AT*)			
✓ Adult	Days	25,550	[41]
✓ Child	Days	3650	[42]

**Table 4:** Oral *RfD* and oral *SF* of HMMs.

Heavy Metal	Oral RfD (mg/kg/day)	SF (mg/kg/day)^−1^	Reference
Arsenic	3 × 10^−4^	1.5	[44]
Barium	2 × 10^−1^	-	[45]
Copper	0.04	-	[46]
Iron	7 × 10^−1^	-	[47]
Manganese	1.4 × 10^−1^	-	[47]
Nickel	0.02	0.84	[46]
Zinc	0.3	-	[46]
Lead	0.0014	8.5 × 10^−3^	[46]

**Table 5:** Carcinogenic risk assessment scale [49].

Risk Level	*TCR* Value	Cancer Risk
1	*TCR* < 10^−6^	Very low
2	10^−6^ < *TCR* < 10^−5^	Low
3	10^−5^ < *TCR* < 10^−4^	Medium
4	10^−4^ < *TCR* < 10^−3^	High
5	*TCR* > 10^−3^	Very high

**Table 6:** Mean values of physicochemical parameters and HMM concentrations.

Parameters	Unit	WRS (*n* = 25)			GW (*n* = 26)			TW (*n* = 49)			WHO [1]	PNSDW [48]
		Mean	SD	CV	Mean	SD	CV	Mean	SD	CV		
Temperature	°C	26.6	3.69	13.9	29.3	1.99	6.78	29.4	1.61	5.48	-	-
pH	-	6.74	0.89	13.2	7.03	0.48	6.78	6.91	1.03	14.9	6.5–9.5	6.5–8.5
EC	μS/cm	51.6	84.6	164	680	735	108	378	286	75.7	-	-
TDS	ppm	19.2	38.8	202	328	367	112	180	143	79.0	-	600
As	mg L^−1^	0.515	1.86	361	0.106	0.19	184	1.05	3.84	365	0.01	0.01
Ba	mg L^−1^	0.027	0.02	70.2	0.025	0.02	60.7	0.023	0.02	80.8	0.70	0.70
Cu	mg L^−1^	0.038	0.08	222	0.025	0.06	250	0.027	0.09	319	2.00	1.00
Fe	mg L^−1^	0.178	0.31	173	0.901	2.93	325	0.138	0.31	225	0.30	1.00
Pb	mg L^−1^	0.371	0.59	158	1.23	3.03	247	0.432	0.80	184	0.01	0.01
Mn	mg L^−1^	0.009	0.004	41.8	0.009	0.01	68.2	0.010	0.01	49.3	0.40	0.40
Ni	mg L^−1^	0.082	0.02	30.5	0.077	0.04	55.2	0.208	0.58	277	0.07	0.07
Zn	mg L^−1^	0.029	0.01	47.7	0.035	0.03	93.6	0.030	0.02	50.7	3.00	5.00

**Table 7:** Trends of HMM concentrations (from highest to lowest) in the DW of Marinduque Province, Philippines.

Water Sources	Trend
WRS	As > Pb > Fe > Ni > Cu > Zn > Ba > Mn
GW	Pb > Fe > As > Ni > Zn > Ba > Cu > Mn
TW	As > Pb > Ni > Fe > Zn > Cu > Ba > Mn

**Table 8:** The mean *CDI* of metals (mg/L) through oral route from water.

Water Sources	HMMs							
	As	Ba	Cu	Fe	Pb	Mn	Ni	Zn
TW (adult)	0.0331	0.0007	0.0009	0.0043	0.0136	0.0003	0.0065	0.0010
GW (adult)	0.0033	0.0008	0.0008	0.0283	0.0386	0.0003	0.0024	0.0011
WRS (adult)	0.0162	0.0008	0.0011	0.0056	0.0117	0.0003	0.0026	0.0009
TW (child)	0.0421	0.0009	0.0011	0.0055	0.0173	0.0004	0.0083	0.0012
GW (child)	0.0042	0.0010	0.0010	0.0360	0.0491	0.0004	0.0031	0.0014
WRS (child)	0.0206	0.0011	0.0015	0.0071	0.0148	0.0004	0.0033	0.0011

**Table 9:** Mean non-CR parameters (*HQ* and *HI*) of HMMs in the water.

Water Sources	*HQ*								*HI*
	As	Ba	Cu	Fe	Pb	Mn	Ni	Zn	
Tap water (adult)	110 *	0.004	0.022	0.006	9.71 *	0.002	0.327	0.003	120 *
GW (adult)	3.14 *	0.004	0.006	0.011	35.0 *	0.002	0.123	0.002	38.2 *
WRS (adult)	30.6 *	0.005	0.015	0.005	7.50 *	0.002	0.129	0.003	38.3 *
Tap water (child)	140 *	0.005	0.028	0.008	12.4 *	0.003	0.416	0.004	153 *
GW (child)	14.2 *	0.005	0.025	0.052	35.1 *	0.003	0.155	0.005	49.5 *
WRS (child)	68.7 *	0.005	0.037	0.010	10.6 *	0.003	0.164	0.004	79.5 *

* Potential high HR to human population.

**Table 10:** Mean total carcinogenic risk parameters (*TCR*) of metals in the domestic water.

Water Sources	*CR*			*TCR*
	As	Pb	Ni	
Tap water (adult)	4.96 × 10^−2^	1.16 × 10^−4^	5.50 × 10^−3^	5.53 × 10^−2^
Groundwater (adult)	1.41 × 10^−3^	4.16 × 10^−4^	2.07 × 10^−3^	3.90 × 10^−3^
WRS (adult)	1.38 × 10^−2^	8.92 × 10^−5^	2.16 × 10^−3^	1.60 × 10^−2^
Tap water (child)	1.50 × 10^−1^	3.32 × 10^−4^	2.35 × 10^−3^	1.53 × 10^−1^
Groundwater (child)	1.80 × 10^−3^	5.29 × 10^−4^	2.63 × 10^−3^	4.96 × 10^−3^
WRS (child)	1.75 × 10^−2^	1.14 × 10^−4^	2.76 × 10^−3^	2.04 × 10^−2^

**Table 11:** NN-PSO simulation results for the *MPI* mapping of domestic water samples.

	Hidden Neurons	No. of Particles	No. of Iterations	Elapsed Time (sec)	MSE	R	
						Validation	Testing
TW	27	10	2000	179.07227	0.00946	0.99943	0.97347
GW	29	6	2000	158.96334	0.00269	0.98592	0.95757
WRS	25	10	2000	118.08149	0.00240	0.99995	0.99063

**Table 12:** NN-PSO simulation results for the *NPI* mapping of domestic water samples.

	Hidden Neurons	No. of Particles	No. of Iterations	Elapsed Time (sec)	MSE	R	
						Validation	Testing
TW	28	2	2000	156.89022	0.004781	0.99111	0.98528
GW	26	8	2000	160.20618	0.001309	0.97545	0.98381
WRS	29	10	2000	162.05585	0.001384	0.99959	0.96495

**Table 13:** NN-PSO simulation results for the *HI* (adults) mapping of domestic water samples.

	Hidden Neurons	No. of Particles	No. of Iterations	Elapsed Time (sec)	MSE	R	
						Validation	Testing
TW	30	5	2000	175.55656	0.00275	0.99856	0.99881
GW	25	5	2000	168.88374	0.00386	0.97732	0.99998
WRS	28	9	2000	124.37414	0.00142	0.99935	0.99994

**Table 14:** NN-PSO simulation results for the *HI* (children) mapping of domestic water samples.

	Hidden Neurons	No. of Particles	No. of Iterations	Elapsed Time (sec)	MSE	R	
						Validation	Testing
TW	28	10	2000	159.46539	0.00585	0.99913	0.99628
GW	30	6	2000	178.66654	0.00237	0.99985	0.99978
WRS	26	3	2000	186.69597	0.00173	0.99637	0.99758

**Table 15:** NN-PSO simulation results for the *TCR* (adult) mapping of domestic water samples.

	Hidden Neurons	No. of Particles	No. of Iterations	Elapsed Time (sec)	MSE	R	
						Validation	Testing
TW	30	7	2000	132.47061	0.00082	0.99628	0.99924
GW	26	7	2000	169.82680	0.00002	0.99950	0.99589
WRS	29	6	2000	169.27427	0.00001	0.99957	0.99629

**Table 16:** NN-PSO simulation results for the *TCR* (children) mapping of domestic water samples.

	Hidden Neurons	No. of Particles	No. of Iterations	Elapsed Time (sec)	MSE	R	
						Validation	Testing
Tap Water	29	7	2000	166.27182	0.00028	0.99871	0.94267
GW	26	7	2000	169.06680	0.00002	0.99873	0.99979
WRS	29	6	2000	130.73092	0.00001	0.97401	0.98393

## Data Availability

All data are contained in the manuscript.
